# Intramedullary nailing for subtrochanteric fracture in autosomal dominant Type II osteopetrosis: Case report of 2 patients

**DOI:** 10.1097/MD.0000000000021648

**Published:** 2020-08-07

**Authors:** Junyoung Kim, Young Chang Park, Hyun-Soo Moon, Woo Sung Do, Kyu Hyun Yang

**Affiliations:** a Department of Orthopedic Surgery, Gangnam Severance Hospital, Yonsei University College of Medicine, Gangnam-gu, Seoul; b Department of Orthopedic Surgery, International St. Mary's Hospital, Catholic Kwandong University College of Medicine, Seo-gu, Incheon, Republic of Korea.

**Keywords:** intramedullary nailing, osteopetrosis, subtrochanteric fracture ;

## Abstract

**Rationale:**

Autosomal dominant type II (AD II) osteopetrosis is a rare inheritable metabolic bone disease characterized by hard but brittle bone and a narrow medullary canal. Intramedullary nailing (IMN) is a difficult but attractive option for the treatment of subtrochanteric fractures in patients with AD II osteopetrosis.

**Patient concerns and diagnosis:**

Two patients with AD II osteopetrosis sustained subtrochanteric fractures after a fall.

**Interventions:**

IMN was performed through the sequential use of instruments such as a 4.9-mm drill bit, small reamer, and larger reamer for over-reaming.

**Outcomes:**

In the first case, IMN left some gap at the fracture site. Dynamization was performed to treat the delayed union at 6 months postoperatively. The fracture healed at 10 months after the dynamization. In the second case, IMN was successful without a gap, and the fracture healed at 8 months.

**Lessons:**

Although IMN is difficult to perform owing to partial obliteration of the medullary canal in AD II osteopetrosis, it can be performed with sequential widening of the medullary canal using various instruments. In addition, the fracture gap should not be left uncorrected during IMN to attain fracture union.

## 1 Introduction

Autosomal dominant type II (AD II) osteopetrosis is a rare inheritable metabolic bone disease caused by mutation in chloride channel 7 genes and osteoclast dysfunction.^[1,2]^ It is characterized by hard but brittle bone, a narrow medullary canal, and a subtrochanteric fracture.^[2]^ Owing to the biomechanical superiority of intramedullary nailing (IMN) in subtrochanteric fracture fixation, IMN is an attractive option. In ordinary IMN, the ball-tip guide wire passes through the open medullary canal without difficulties, facilitating the reduction and reaming processes. Although IMN is the treatment of choice for subtrochanteric fractures, medullary canal deformities with obstruction and osteopetrotic bone hardness preclude ordinary IMN.^[3]^

Although anecdotal case reports have described the surgical management of osteopetrotic subtrochanteric femoral fracture, the treatment strategies varied among them.^[4–6]^ In the existing case reports in the literature, IMN was rarely performed and led to unsatisfactory results due to insufficient reaming of the medullary canal. To address these issues, we report an IMN surgical technique step-by-step for patients with AD II osteopetrotic subtrochanteric fracture.

## 2 Case Report

### 2.1 Case 1

**Figure 1:**
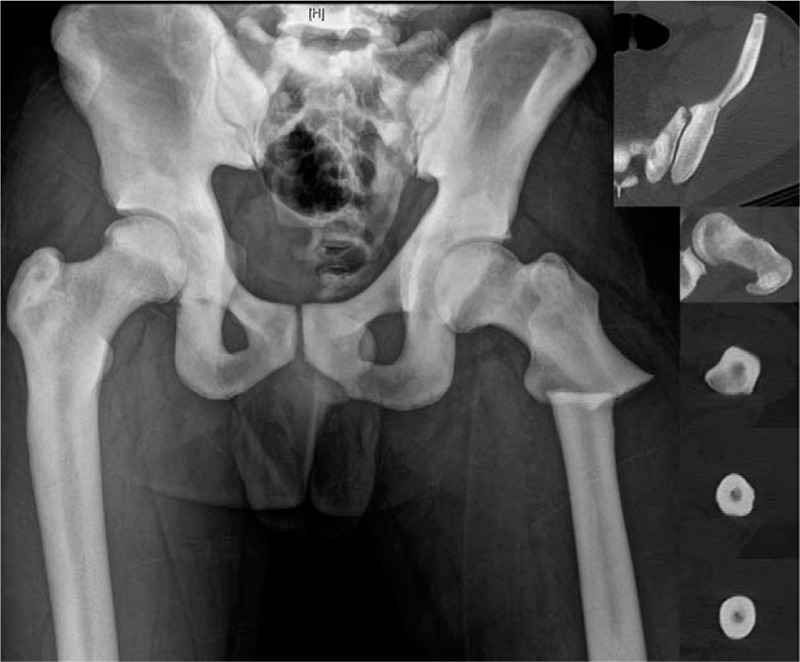
Typical radiography and computed tomography images of the pelvis and femur in a case of autosomal type II osteopetrosis. Generalized osteosclerosis with “bone-within-bone” structures are shown. Inlets, axial computed tomography images matched at the level of the femur.

**Figure 2:**
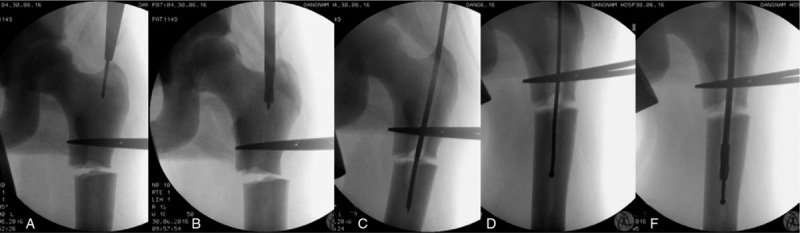
Sequential processes of medullary canal reaming. A. After placement of the 3.2-mm threaded guide pin on the starting point, the entry point was double-checked in the anteroposterior and axial views. B. An 8-mm starting reamer was used to enlarge the entry site for a few centimeters along the guide pin. C. A 4.9-mm-long drill bit was used to enlarge the remnant intramedullary canal. D. After enlarging the remnant medullary canal, a ball-tip guide wire could pass the canal. F. Reaming was started from 7 mm and completed at 11.5 mm in 0.5-mm increments.

A 26-year-old man who was diagnosed as having AD II osteopetrosis at 10 years of age sustained a displaced subtrochanteric fracture without comminution after a fall. Radiography of the pelvis and bilateral femurs revealed generalized osteosclerosis with “bone-within-bone” structures and narrow medullary canals. As the intramedullary canal was traceable on plain radiography and computed tomography, we planned an IMN (Fig. 1). The patient was positioned on the fracture table under general anesthesia. The proximal fragment was derotated internally using long curved hemostatic forceps to obtain a good anteroposterior hip image.^[7]^ A longitudinal skin incision was made from the palpable tip of the greater trochanter and extended proximally for approximately 5 cm. The entry point was prepared just medial to the tip of the greater trochanter. After placement of the 3.2-mm threaded guide pin on the starting point, the entry point was double-checked in the anteroposterior and axial views (Fig. 2). This 3.2-mm guide pin could enter only a few centimeters because of the bone hardness in the trochanteric area. An 8-mm starting reamer was used to enlarge the entry site for a few centimeters along the guide pin. We then used a 4.9-mm drill bit to open the remnant medullary canal in the proximal fragment. A 4.9-mm drill bit was replaced with a 3.2-mm guide pin. The proximal fragment was reamed with a 14.5-mm tapered (trochanteric) reamer to accommodate a 13-mm nail body.

**Figure 3:**
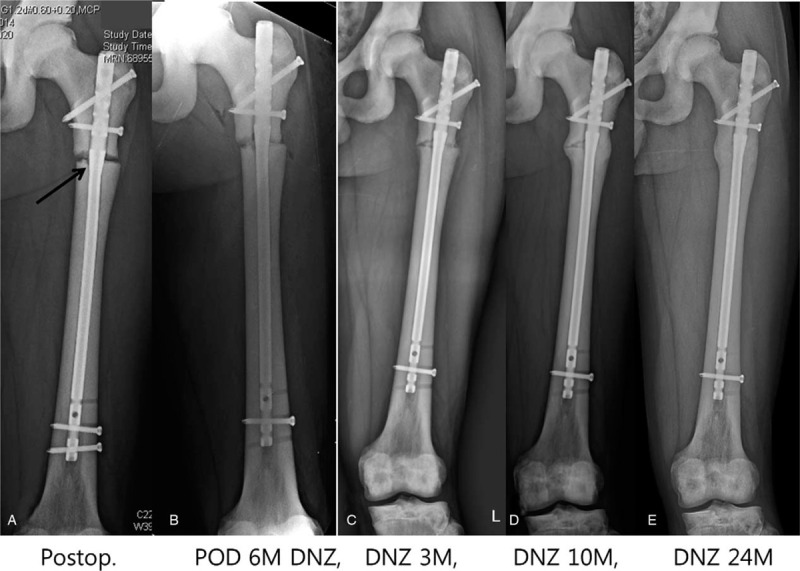
A Subtrochanteric fracture in a 26-year-old man with autosomal dominant type II osteopetrosis. A. A subtrochanteric fracture was treated with intramedullary nailing. However, a gap in the fracture site occurred because of contact between the transitional part of the nail and the distal fragment (arrow). B. Delayed union was evident at 6 months after the operation, and dynamization (DNZ) was performed. C. Callus formation was observed at 3 months after the DNZ. D. The fracture attained union 10 months after the DNZ. F. The final radiograph 24 months after the DNZ shows complete union and remodeling of the callus. POD, postoperative day.

We continued to ream the distal fragment with a 4.9-mm drill bit that followed the remnant intramedullary canal. Breakage of the drill bit makes IMN impossible, so it must be advanced with special care and kept in the center of the canal. After enlarging the remnant medullary canal, a ball-tip guide wire could pass the canal. Reaming was started at 7 mm and finished at 11.5 mm in 0.5-mm increments. The reamer must advance slowly in maximum revolutions per minute with frequent saline irrigation. This procedure was repeated every 2 cm until it reached the distal one-third of the medullary canal. Over-reaming of 1.5–2 mm is necessary to advance the nail manually. Forceful impaction on the nail with a hammer may break the brittle bone; thus, it is not recommended. After nail insertion, proximal interlocking screws were inserted through the targeting guide without difficulty. However, the insertion of a distal interlocking screw was quite difficult by free hand technique owing to the hardness of the osteopetrotic bone, which caused skidding of the drill bit. The postoperative radiograms showed that we made a mistake and left a gap in the fracture site. It was caused by contact between the transitional part of the nail and the distal fragment (the arrow in Fig. 3A). The proximal part of the distal fragment was not reamed enough to accept the thickened portion (shaft-body transitional part) of the nail. As delayed union was evident at 6 months postoperatively, a dynamization procedure was performed (Fig. 3B, C). We exposed the proximal end of the nail, assembled a targeting guide, and removed the proximal and distal interlocking screws except for the one in the distal oval hole. The nail-targeting guide complex was pulled back until the fracture gap was closed. This movement also moved the distal locking screw to the dynamic locking mode (screw moved to the distal end of the oval hole). We then reinserted the proximal interlocking screws. Callus started to form 3 months after the dynamization. Bony union was achieved 10 months after the dynamization (16 months after the injury), and remodeling was completed 30 months after the injury (Fig. 3D, E).

### 2.2 Case 2

**Figure 4:**
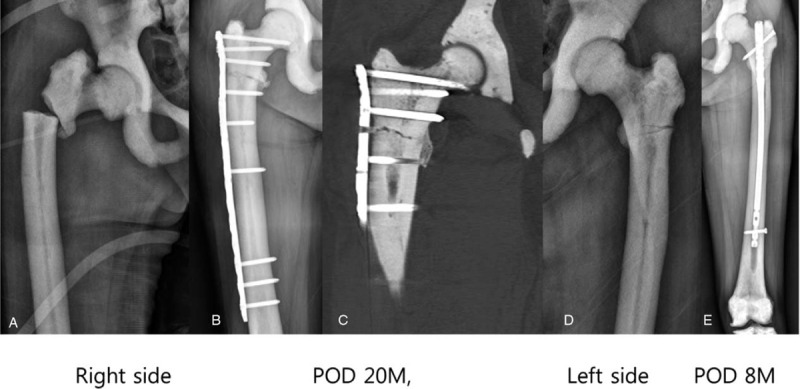
Bilateral subtrochanteric fractures in a 70-year-old woman with autosomal dominant type II osteopetrosis. A and D. A transverse subtrochanteric fracture at the lower level of the lesser trochanter in the right femur and incomplete fracture in the left femur are shown. B, C. Open reduction and plate fixation were performed on the right side. The peripheral callus is visible on plain radiography and computed tomography at 20 months postoperatively, but the fracture line remained because of delayed bone remodeling. E. The incomplete fracture line disappeared 8 months after prophylactic nailing on the left side.

A 70-year-old woman was transferred to the emergency department because of right hip pain after a fall. A transverse subtrochanteric fracture was identified at the lower level of the lesser trochanter, without comminution, in the right femur (Fig. 4A). An incomplete subtrochanteric fracture was also identified at the same level in the left femur (Fig. 4D). The incomplete fracture started from the lateral femoral cortex and extended to the medial cortex just like an atypical femoral fracture. Prophylactic IMN was indicated on the left side and performed in the dynamic locking mode uneventfully using the technique introduced earlier. The nail was inserted in external rotation to overcome the femoral bowing.^[8]^ The fracture achieved complete union and remodeled 8 months postoperatively (Fig. 4E). However, a short proximal fragment and associated femoral bowing precluded the IMN on the right side, and open reduction and internal fixation with a locking plate and screws were performed (Fig. 4B). A periosteal callus was formed at the periphery of the fracture site, but the main fracture line did not disappear even at 20 months postoperatively, probably owing to the delayed callus remodeling in osteopetrosis (Fig. 4C).

## 3 Discussion

Deformity of the long bone and an associated deformity of the medullary canal are usually contraindications for IMN. However, IMN with or without an osteotomy is sometimes the only reliable option in cases of heritable bone disease such as osteogenesis imperfecta and X-linked hypophosphatemic rickets.^[9,10]^ In AD II osteopetrosis, the long bones are usually straight but have only traceable amounts of medullary canal. Despite the narrow medullary canal, closed IMN was possible owing to the sequential use of instruments that could keep the reamers at the center of the partially obliterated medullary canal.

Technological advances have enabled IMN in the narrow medullary canal of the femur. We used 10-mm-diameter nails in both cases to decrease the amount of reaming. However, we needed to ream the proximal fragment with a 14.5-mm trochanter reamer to accommodate a 13-mm nail body. Reaming was insufficient in the proximal part of the distal fragment to accept the thickened transitional portion of the nail in case 1. It pushed on the distal fragment, causing a gap at the subtrochanteric area. No sign of healing was observed until 6 months. To avoid this mistake, the proximal part of the femur must be reamed sufficiently with a trochanteric reamer. The fracture gap must be checked and should not be left uncorrected after nail insertion by hand. Bone union was achieved without deformity after the dynamization. We found similar mistakes in the literature, such as bursting of the fracture site due to insufficient reaming for the nail body during cephalomedullary nailing.^[11]^ Kumbaraci et al reported bilateral subtrochanteric femoral fractures in AD II osteopetrosis, which were treated with open cephalomedullary nailing in 21-year-old female patient. They experienced bursting of the distal fragment due to the thick nail body even after open nailing. Cephalomedullary nails have a larger nail body (range, 15.5–16.5 mm) than femoral nails, so they are more difficult to place sufficiently into the proximal femur. The bilateral subtrochanteric fractures healed with a callus on both sides but without evidence of remodeling at 12 months postoperatively.

Owing to bone hardness and reaming difficulties, many surgeons prefer open reduction and internal fixation with a plate and screws.^[3,4]^ Although callus formation is grossly not retarded in AD II osteopetrosis, a thick locking or non-locking plate must be kept in place for a long time owing to defects in osteoclast and bone remodeling (Fig. 4C). The risks of soft tissue irritation and fixation failure in the subtrochanteric area are higher in cases of extramedullary implants than in cases of IMN.^[12,13]^ Thus, closed IMN with sequential enlargement of the narrow medullary cavity by using various instruments is an attractive option for skilled surgeons in the treatment of subtrochanteric fractures in AD II osteopetrosis.

## 4 Conclusion

Although IMN is difficult to perform because of partial obliteration of the medullary canal in AD II osteopetrosis, it can be performed by sequential widening of the medullary canal using various instruments. In addition, the fracture gap should not be left uncorrected during IMN to attain fracture union.

## Acknowledgments

We thank John Hoon Rim, M.D., and Heon Yung Gee, M.D., Ph.D., for identifying the gene mutations in these 2 patients with autosomal dominant type II osteopetrosis.

## Author contributions

Junyoung Kim, Substantial contributions to the conception or design of the work; or the acquisition, analysis, or interpretation of data for the work/Final approval of the version to be published.

Young Chang Park, Drafting the work or revising it critically for important intellectual content/ Final approval of the version to be published.

Hyun-Soo Moon, Substantial contributions to the conception or design of the work; or the acquisition, analysis, or interpretation of data for the work.

Woo Sung Do, Substantial contributions to the conception or design of the work; or the acquisition, analysis, or interpretation of data for the work.

Kyu Hyun Yang, Substantial contributions to the conception or design of the work; or the acquisition, analysis, or interpretation of data for the work/Drafting the work or revising it critically for important intellectual content/Final approval of the version to be published.
